# Phosphorus Alters the Metabolism of Sugars and Amino Acids in Elite Wheat Grains

**DOI:** 10.3390/plants14203152

**Published:** 2025-10-13

**Authors:** Jialian Wei, Xiangchi Zhang, Gang Li, Kaiyong Fu, Mei Yan, Cheng Li, Chunyan Li

**Affiliations:** 1 Agricultural College, Shihezi University, Key Laboratory of Oasis Eco-Agriculture, Xinjiang Production and Construction Corps, Shihezi 832003, China; m18386191910@163.com (J.W.); 17699537028@163.com (X.Z.); ligang@xjshzu.com (G.L.); fucareu1993@163.com (K.F.); yanmei1029@163.com (M.Y.); 2 Xinjiang Academy of Agricultural and Reclamation Sciences, Shihezi 832000, China

**Keywords:** carbohydrate allocation, phosphorus, starch granule-associated proteomes, wheat

## Abstract

Phosphorus supply significantly influences starch and amino acid accumulation in wheat grains, yet the mechanisms coordinating sugar–amino acid metabolic crosstalk under differential phosphorus availability remain elusive. To address this knowledge gap, we conducted a controlled trial on phosphorus supplementation using wheat (Triticum aestivum L. cv. Xindong 20) with three treatments: P0 (0 kg·ha^−1^, phosphorus deficiency), LP (105 kg·ha^−1^, normal phosphorus), and HP (210 kg·ha^−1^, phosphorus excess). Seed samples were collected at 7, 14, and 21 days post-anthesis (DPA). This design enabled a systematic analysis of how phosphorus availability modulates the metabolic relationship between amino acids and sugars during grain development. Proteomic profiling of starch granule-associated proteins (SGAPs) demonstrated that wheat reprograms carbohydrate allocation in response to phosphorus availability. Notably, differentially expressed proteins (DEPs) exhibited tissue-specific regulation patterns: pericarp-localized DEPs were predominantly up-regulated, whereas endosperm-associated DEPs showed down-regulation under phosphorus modulation. Mechanistically, phosphorus application triggered accelerated starch catabolism in the pericarp (Pe) concomitant with enhanced starch anabolism in the endosperm (En), thereby altering the temporal dynamics of starch granule development. These findings elucidate key regulatory patterns of phosphorus nutrition in wheat grain metabolism, establishing a biochemical framework for the optimization of starch quality parameters. The identified phosphorus-responsive metabolic networks reveal pivotal mechanisms that support the development of precision breeding strategies and phosphorus-efficient cultivation practices. This research offers novel pathways to simultaneously improve both grain yield and nutritional quality in wheat production systems.

## 1. Introduction

Phosphorus is a vital macronutrient for plants, playing a central role in carbon metabolism, energy transfer, sugar–starch interconversion, and nutrient translocation. However, its low availability in most soils severely constrains plant growth and physiological functionality [1,2]. While the fundamental importance of phosphorus in plant metabolism is well established, critical uncertainties remain regarding its specific regulatory roles in wheat grain development. In particular, it is still unclear how phosphorus availability modulates the metabolic crosstalk between sugars and amino acids in developing wheat caryopses, and how such interactions ultimately influence starch and protein composition. This knowledge gap directly impacts agronomic practice, as the optimal phosphorus application rate for balancing wheat yield and grain quality—without causing resource waste or environmental pollution—has yet to be determined.

The development of starch granules in wheat caryopses is a spatiotemporally regulated process. A- and B-type starch granules are formed in the endosperm under strict temporal control [3], while starch in the pericarp is transient and serves as a temporary carbon reserve. It is known that pericarp starch accumulation peaks around 5 days post-anthesis (DPA) and declines markedly by 11 DPA [4,5]. This degraded starch is likely converted into soluble sugars that may support subsequent starch biosynthesis in the endosperm [4,6]. By contrast, endosperm starch serves as a long-term storage reserve [7]. A-type granules initiate at 4–14 DPA, B-type granules emerge around 10–16 DPA, and smaller C-granules appear near 21 DPA [8]. Beyond granule morphology, starch composition and functionality are strongly influenced by starch granule-associated proteins (SGAPs). Although SGAPs are present at low abundance, they exert a disproportionate impact on starch physicochemical properties—often greater than that of storage proteins [9,10]. SGAPs interact directly with starch and participate in regulating resistant starch biosynthesis by modulating amylose content, amylose-lipid complex formation, and amylopectin structure [11,12].

Sugar and amino acid metabolisms are critical metabolic pathways in plants and are closely linked to the growth, development, and quality of wheat. Sugar serves as a substrate for starch synthesis, providing not only the energy and metabolic intermediates essential for the growth and development of wheat but also influencing its flavor and processing characteristics [13]. Amino acids are building blocks of protein synthesis and are directly associated with the nutritional quality of wheat [14]. Amino acids and sugars are integral to the synthesis of various substances, which are crucial for the normal growth and development of organisms. Their degradation processes complement one another, providing the energy necessary to meet the energy demands of organisms [15,16]. In different cellular compartments, coordination of sugar and amino acid metabolic pathways plays a crucial role in promoting mutual regulation between these pathways [16]. Particularly, in the context of carbon–nitrogen interactions, the availability of phosphorus serves as an important regulatory factor in this process. The synthesis and metabolism of sugars and amino acids are significantly influenced by phosphorus supply. Research has demonstrated that phosphorus is not only a key component of nucleic acids, phospholipids, and ATP in plants, but also directly affects carbon metabolism and amino acid synthesis pathways [17]. To ensure normal functioning of cellular activities, plants have evolved glycolytic metabolic bypasses that do not rely on inorganic phosphate (Pi), allowing them to circumvent steps that require phosphorus. This adaptation helps mitigate the adverse effects of phosphorus deficiency on plant health [2,18,19]. Under phosphorus-deficient conditions, carbohydrate metabolism is realigned to ensure energy acquisition and to meet basic metabolic needs. Phosphorus limitation induces significant alterations in the carbohydrate metabolic pathways of plants, resulting in sugar accumulation and the activation of stress response mechanisms [20]. Additionally, the synthesis of amino acids within the plant is inhibited, a process that is closely linked to sugar utilization [21]. However, the tissue-specific dynamics of these responses in wheat pericarp and endosperm remain poorly characterized.

In summary, while phosphorus is recognized as a central regulator of wheat metabolism, the mechanisms by which it orchestrates sugar and amino acid interactions in different grain tissues—and how these processes impact starch and protein quality—are not yet fully understood. To address these gaps, this study aims to systematically investigate the influence of varying phosphorus supply on the metabolic crosstalk between sugars and amino acids during wheat grain development. We hypothesize that phosphorus availability differentially regulates sugar and amino acid metabolism in pericarp and endosperm tissues, and that these responses are mediated in part by SGAPs, ultimately defining starch physicochemical properties and grain nutritional value. Our specific objectives are: (1) to characterize the tissue-specific responses of sugar and amino acid metabolism to phosphorus application using integrated physiological and transcriptomic analyses; (2) to elucidate the role of SGAPs in mediating phosphorus-dependent effects on starch biosynthesis and functionality; and (3) to decipher the biosynthetic mechanisms that coordinate carbon and nitrogen metabolism under different phosphorus regimes. The findings are expected to provide a theoretical foundation for optimizing phosphorus fertilization strategies aimed at enhancing both yield and grain quality in an environmentally sustainable manner.

## 2. Results

### 2.1. Phosphorus Supply Levels Affected Morphology of Starch Granules

**Figure 1:**
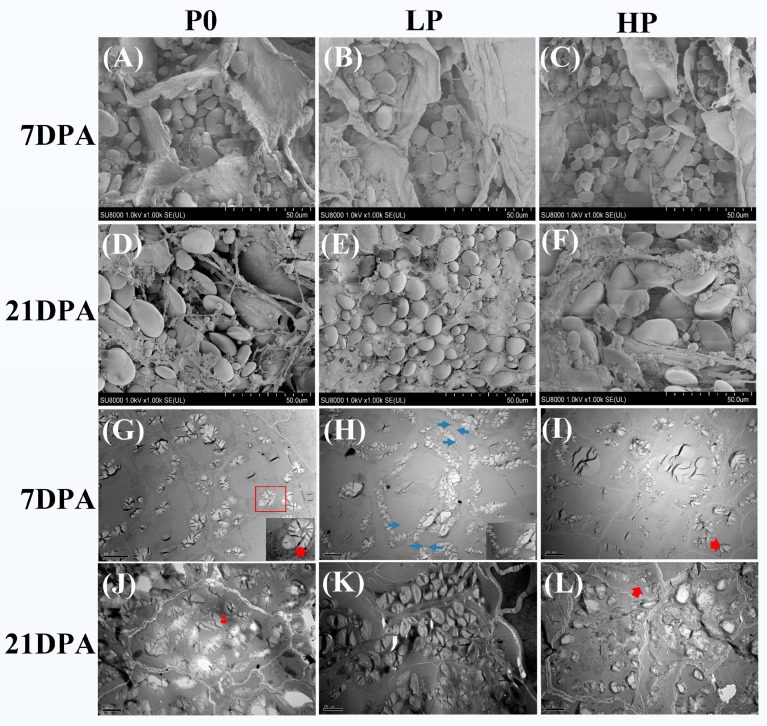
Phosphorus-dependent ultrastructural morphology of wheat starch granules. (**A**–**C**) Scanning electron micrographs (SEM) of pericarp starch granules at 7 days post-anthesis (DPA); (**D**–**F**) SEM visualization of endosperm starch granules at 21 DPA; (**G**–**I**) Transmission electron micrographs (TEM) of endosperm starch granules at 7 DPA (In (**G**), the scale bar in the lower right corner is 2 μm; In (**H**), the scale bar in the lower right corner is 5 μm); (**J**–**L**) TEM analysis of endosperm starch granules at 21 DPA. Phosphorus treatments: P0 (0 kg·ha^−1^, no phosphorus); LP (105 kg·ha^−1^, normal phosphorus) and HP (210 kg·ha^−1^, excessive phosphorus). Consistent nomenclature is maintained throughout subsequent analyses. The equatorial groove, denoted by the blue arrow, while the concurrent expansion of the amyloplast envelope, highlighted by the red arrow and box. Data represent mean values ± SE (*n* = 3 biological replicates). Statistical significance was determined by *t*-tests.

The SEM results showed that the number of B-type starch granules (diameter < 10 μm) in the pericarp of the HP group significantly increased at 7 DPA compared with that of the P0 group, with the smaller granules primarily exhibiting a spherical shape and the larger ones exhibiting an ellipsoidal shape (Figure 1A,C). In contrast, at 21 DPA, the number of B-type starch granules in the endosperm of the HP group significantly reduced compared with that of the P0 group, with the smaller granules predominantly attached to the larger ones (Figure 1D,F). Conversely, the LP group demonstrated an opposite change (Figure 1B,E).

TEM revealed distinct developmental patterns in starch granules at 7 DPA. At this stage, starch granules were in their initial developmental stage, predominantly localized near the cell wall (Figure 1G–I). Amyloplasts in all groups contained monogranular starch bodies, which exhibited either spherical or spindle-shaped morphologies. Notably, the LP and HP groups displayed a significantly higher proportion of spindle-shaped starch granules compared to P0 controls. A striking feature observed in the LP group was the presence of equatorial grooves (indicated by blue arrows in Figure 1G) at both poles of the spindle-shaped granules. In contrast, in the P0 group, individual starch granules were enveloped by a membrane, while the entire complex of starch granules was surrounded by the membrane of the amyloplast, with the middle membrane being clearly visible (indicated by the red box) (Figure 1I).

At 21 DPA, endosperm cells exhibited continued proliferation of amyloplasts, marked by increased numerical density and volumetric expansion. Vacuolar structures were fully assimilated, with amyloplasts existing as discrete singular granules (Figure 1G–L). Distinct from the loosely organized starch granules observed in P0 and HP groups, LP-treated specimens displayed densely packed starch matrices, exhibiting negligible intergranular or interplastid spaces (Figure 1K). Notably, nascent microgranules were observed developing within plasma membrane invaginations of larger starch bodies, as demarcated by the red arrow.

Regarding the starch granule size distribution, compared with the P0 treatment, the LP and HP treatments were more conducive to the generation of smaller starch granules (Figure S1A). In the early stage, both the P0 and HP groups promoted the formation of type B starch granules.

Throughout the entire filling period, the LP group significantly facilitated the formation of long-chain starch molecules. Notably, the LP group significantly increased the proportion of DP19–34 (B1-chains) and DP > 35 (B2-chains), while reducing the proportion of A-chains (DP6–18) (Figure S1B).

### 2.2. Phosphorus Supply Levels Affected the Content of Starch and Soluble Sugars in Wheat Grains

**Figure 2:**
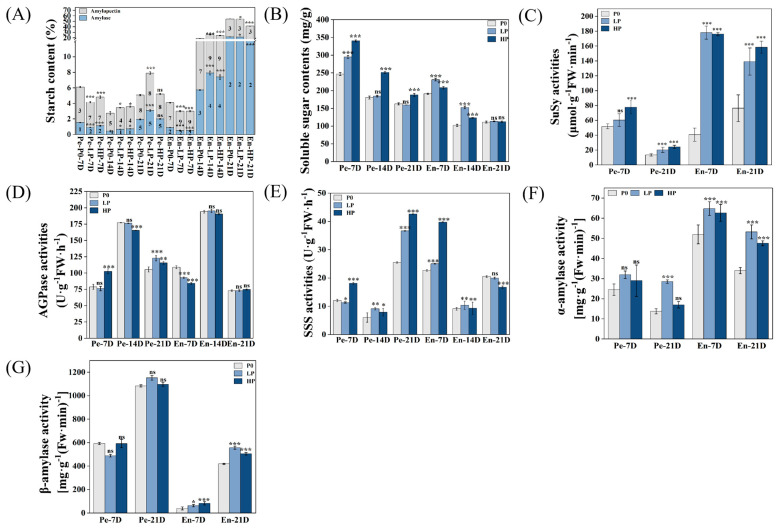
Dynamics of starch metabolism parameters in wheat pericarp and endosperm under differential phosphorus regimes. (**A**) Temporal variations in starch composition during grain development (7, 14, and 21 DPA). Numerical values denote amylose-to-amylopectin ratios, with total starch content representing the combined mass of both fractions. Superscript asterisks indicate significant differences in amylopectin content, while subscript asterisks denote amylose content variations. (**B**) Soluble sugar accumulation patterns across developmental stages. (**C**–**F**) Enzymatic activity profiles of key starch metabolic enzymes: (**C**) Sucrose synthase at critical developmental phases; (**D**) ADP-glucose pyrophosphorylase temporal activity; (**E**) Soluble starch synthase activation patterns; (**F**) α-amylase and (**G**) β-amylase hydrolase activities. Pe, pericarp; En, endosperm. Phosphorus treatments: P0 (0 kg·ha^−1^, no phosphorus); LP (105 kg·ha^−1^, normal phosphorus) and HP (210 kg·ha^−1^, excessive phosphorus). Data represent mean values ± SE (*n* = 3 biological replicates). Statistical significance was determined by *t*-tests (* *p* < 0.05, ** *p* < 0.01, *** *p* < 0.001; ns = not significant).

At 14 DPA, the total starch content in the pericarp and endosperm of the LP and HP groups significantly increased compared with that of the P0 group. At 21 DPA, the total starch content in the pericarp of the LP group significantly increased, whereas no significant change was observed in the HP group. Notably, at 7 and 14 DPA, the amylose/amylopectin ratio in the pericarp and endosperm of the LP and HP groups varied significantly compared with that of the P0 group (Figure 2A). The soluble sugar content in the pericarp of the HP group increased significantly at 7, 14, and 21 DPA, but the soluble sugar content of the LP group exhibited a significant increase only at 7 DPA, compared with that of the P0 group. Regarding the endosperm, the soluble sugar content of the LP and HP groups significantly increased at 7 and 14 DPA compared with that of the P0 group, but no significant difference was observed at 21 DPA (Figure 2B).

The activity of SuSy in the pericarp and endosperm of the LP and HP groups significantly increased compared with that of the P0 group, with SuSy activity in the pericarp being lower than that in the endosperm (Figure 2C). Additionally, the activity of AGPase in the pericarp and endosperm peaked at 14 DPA, contrasting with that observed at 7 and 21 DPA. That is, the activity of AGPase in the pericarp was more sensitive to phosphorus supply. At 7 and 21 days post-anthesis (DPA), the activity of ADP-glucose pyrophosphorylase (AGPase) in the pericarp significantly increased compared to the P0 group under high pressure (HP), while it decreased under low pressure (LP) at 14 DPA. In contrast, the AGPase activity in the endosperm significantly decreased compared to the P0 group under both HP and LP at 7 DPA; however, no significant changes were observed in the endosperm at 14 and 21 DPA (Figure 2D). In the pericarp, the activity of SSS of the LP group significantly decreased at 14 and 21 DPA. In the endosperm, the activity of SSS significantly increased after phosphorus application at 7 and 14 DPA, but decreased at 21 DPA (Figure 2E).

At 7 and 21 DPA, the activity of α-amylase in the endosperm was higher than that of the pericarp. The activity of α-amylase in the pericarp and endosperm of the LP and HP groups increased compared with that of the P0 group, with the highest activity observed in the LP group (Figure 2F). However, the activity of β-amylase in the pericarp was higher than that of the endosperm after phosphorus application, with the exception of that of the LP group at 7 DPA, which was lower than that of the P0 and HP groups. All other cases showed higher activity compared with the P0 group (Figure 2G).

### 2.3. Phosphorus Supply Levels Affected Amino Acid Content of Wheat Grains

**Figure 3:**
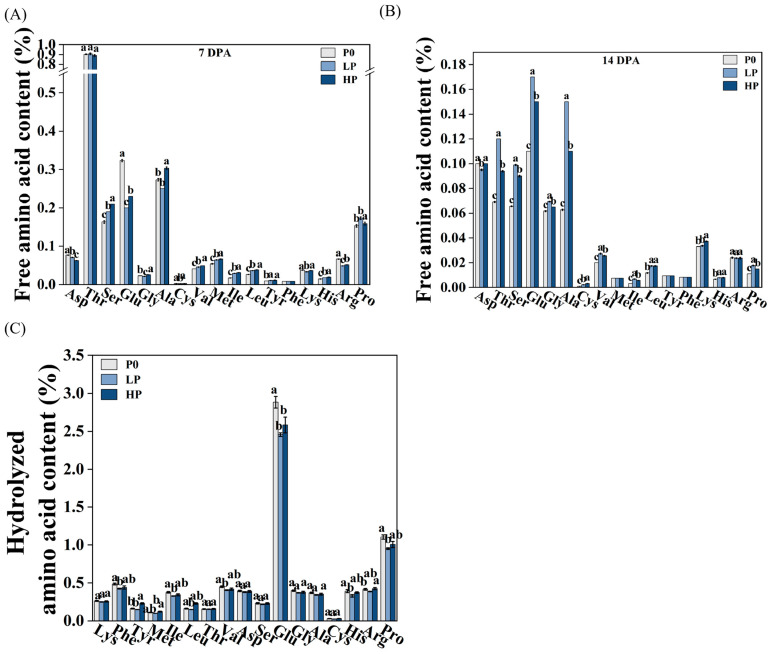
Phosphorus-mediated regulation of amino acid accumulation during grain development. (**A**,**B**) Free amino acid concentration profiles in wheat grains under differential phosphorus regimes at 7 (**A**), 14 (**B**), and hydrolyzed amino acid concentration at 35 DPA (**C**). Within each panel, lowercase letters denote statistically distinct means (*p* < 0.05) between phosphorus treatments, as determined by Fisher’s protected least significant difference (LSD) post hoc test following ANOVA (*n* = 3 biological replicates). Phosphorus treatments: P0 (0 kg·ha^−1^, no phosphorus); LP (105 kg·ha^−1^, normal phosphorus) and HP (210 kg·ha^−1^, excessive phosphorus).

At 7 DPA, compared to the P0 group, both LP and HP groups significantly increased the content of free amino acids, including serine (Ser), valine (Val), methionine (Met), isoleucine (Ile), leucine (Leu), tyrosine (Tyr), and histidine (His). In contrast, the content of glutamic acid (Glu) was significantly reduced. At 14 DPA, LP and HP groups again significantly increased the levels of amino acids such as threonine (Thr), Ser, Glu, glycine (Gly), alanine (Ala), cysteine (Cys), valine (Val), Ile, Leu, His, and proline (Pro) (Figure 3A,B). However, at maturity (35 DPA), the nutritional composition of amino acids in wheat grains under the P0 group was higher than that under LP and HP for all amino acids except for Tyr and Met. Additionally, the content of lysine (Lys) and Thr showed no significant difference (Figure 3C).

### 2.4. Phosphorus Supply Levels Affected the Phosphorus Content in Wheat Grains

**Figure 4:**
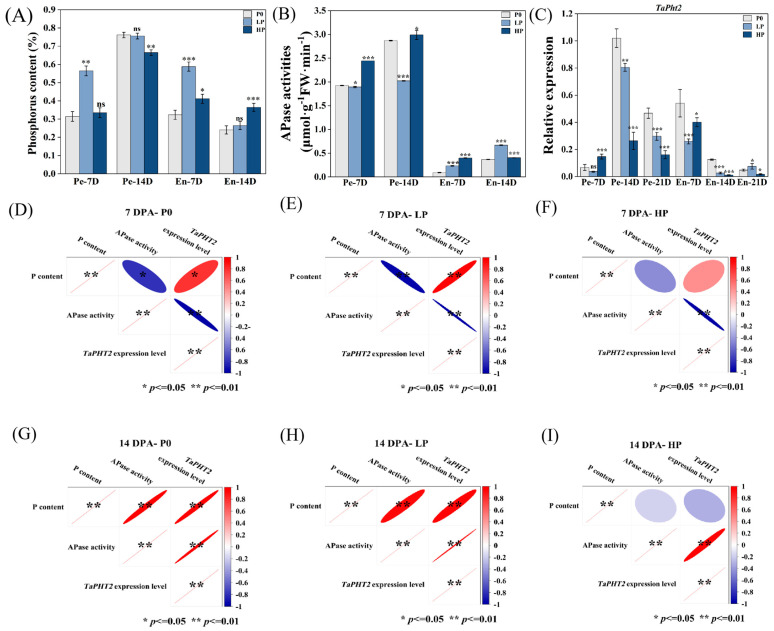
Interparameter correlations under differential phosphorus regimes. (**A**) Phosphorus content at 7 and 14 DPA; (**B**) Acid phosphatase activity dynamics; (**C**) Transcript abundance of the phosphorus transporter gene *TaPht2*. Phosphorus treatments: P0 (0 kg·ha^−1^, no phosphorus); LP (105 kg·ha^−1^, normal phosphorus) and HP (210 kg·ha^−1^, excessive phosphorus). Data represent mean values ± SE (*n* = 3 biological replicates). Statistical significance was determined by *t*-tests (* *p* < 0.05, ** *p* < 0.01, *** *p* < 0.001; ns = not significant). (**D**–**I**) Pearson correlation matrices evaluating relationships between phosphorus content, *TaPht2* expression, and APase activity across phosphorus treatments at 7 and 14 DPA. Asterisks denote significance levels of Pearson correlation coefficients: * *p* ≤ 0.05, ** *p* ≤ 0.01, Non-significant correlations *p* > 0.05.

At 7 and 14 DPA, the APase activity in the pericarp was higher than in the endosperm. In the pericarp, the APase activity of the LP group decreased significantly compared to the P0 group, while the HP group showed a significant increase. In the endosperm, both LP and HP groups had higher APase activity than the P0 group (Figure 4A). The expression of *TaPHT2* in the pericarp was higher in the HP group than in the LP and P0 groups, with the P0 group showing higher expression than the LP and HP groups (Figure 4B). At 7 DPA, the phosphorus content in the pericarp and endosperm of the LP group increased significantly compared to the P0 group, while no significant change occurred in the HP group. At 14 DPA, no significant difference in pericarp phosphorus content was observed between the LP and P0 groups, but the HP group had decreased pericarp phosphorus and increased endosperm phosphorus compared to the P0 group (Figure 4C). APase activity and *TaPHT2* expression showed different correlations with phosphorus content, with the exception of a negative correlation between TaPHT2 expression and phosphorus content in the HP group at 14 DPA (Figure 4D–I).

### 2.5. Phosphorus Supply Levels Affected the Metabolic Characteristics of Starch Granule-Associated Proteins in Wheat Pericarp and Endosperm

Differential expression analysis reveals that the quantity of DEPs varies across different samples. Notably, the number of DEPs in the pericarp exceeds that found in the endosperm (Figure S2).

**Figure 5:**
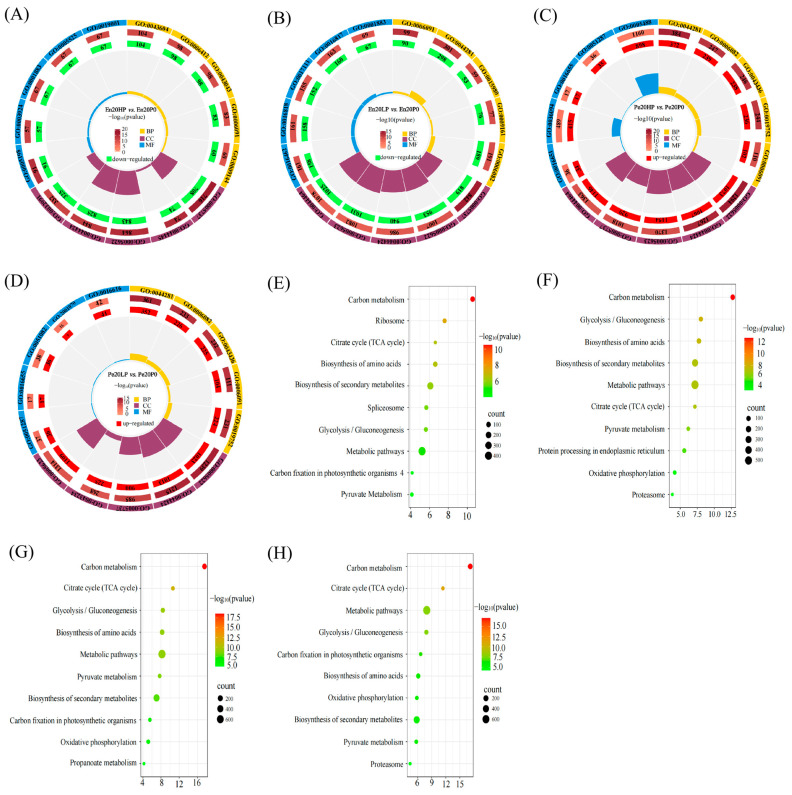
Functional annotation and pathway enrichment of phosphorus-responsive differentially expressed proteins (DEPs). (**A**–**D**) Gene Ontology (GO) categorization of DEPs across tissue-specific phosphorus treatments, with color-coding corresponding to ontology domains: yellow (biological processes, BP), purple (cellular components, CC), and blue (molecular functions, MF). Radial plots display: First annulus: Top five enriched GO terms; Second annulus: DEP count per term (bar length proportional to enrichment magnitude); Third annulus: Differential regulation status (red: up-regulated; green: down-regulated; segment area proportional to protein count). (**E**–**H**) Kyoto Encyclopedia of Genes and Genomes (KEGG) pathway analysis. Node size correlates with metabolite-pathway association count. Comparative groups: (**A**,**E**) Endosperm excessive-phosphorus vs. endosperm no-phosphorus (En20HP vs. En20P0); (**B**,**F**) Endosperm normal-phosphorus vs. no-phosphorus (En20LP vs. En20P0); (**C**,**G**) Pericarp excessive-phosphorus vs. no-phosphorus (Pe20HP vs. Pe20P0); (**D**,**H**) Pericarp normal-phosphorus vs. no-phosphorus (Pe20LP vs. Pe20P0).

GO analysis was performed on the significantly DEPs under varying phosphorus supply levels to better understand the protein response of Xindong 20. The results showed that DEPs in En20HP vs. En20P0 and En20LP vs. En20P0 were significantly down-regulated across various metabolic processes (Figure 5A,B). In contrast, DEPs in Pe20HP vs. Pe20P0 and Pe20LP vs. Pe20P0, enriched in small molecule metabolism and NAD binding, exhibited significant up-regulation (Figure 5C,D).

KEGG analysis revealed that the DEPs in the endosperm and pericarp starch granule-associated proteomes were significantly enriched in carbon metabolism, particularly within metabolic pathways (Figure 5E–H). A comparison of DEPs in En20HP vs. En20P0 and En20LP vs. En20P0 showed that DEPs in En20HP vs. En20P0 were enriched in ribosome, spliceosome, and carbon fixation in photosynthetic organisms, while En20LP vs. En20P0 did not show such enrichments (Figure 5E,F). In the LP group, the enrichment of protein processing in the endoplasmic reticulum, oxidative phosphorylation, and proteasomes indicated increased cellular dependence on protein folding and energy production, reflecting adaptation to environmental changes. The DEPs in Pe20HP vs. Pe20P0 were mainly enriched in propionate metabolism, while this was not significant in Pe20LP vs. Pe20P0. Additionally, the proteasome was enriched in DEPs from Pe20LP vs. Pe20P0 but not in Pe20HP vs. Pe20P0 (Figure 5G,H). In the HP group, pathways like the tricarboxylic acid (TCA) cycle, glycolysis, and amino acid biosynthesis were significantly enhanced, while in the LP group, metabolic activity remained relatively stable. These findings suggest that carbon metabolism is a key process for differentially expressed SGAPs in the endosperm and pericarp responding to phosphorus supply, with distinct metabolic focuses between HP and LP conditions.

### 2.6. Phosphorus Supply Levels Affected on the Redistribution of Carbohydrates

**Figure 6:**
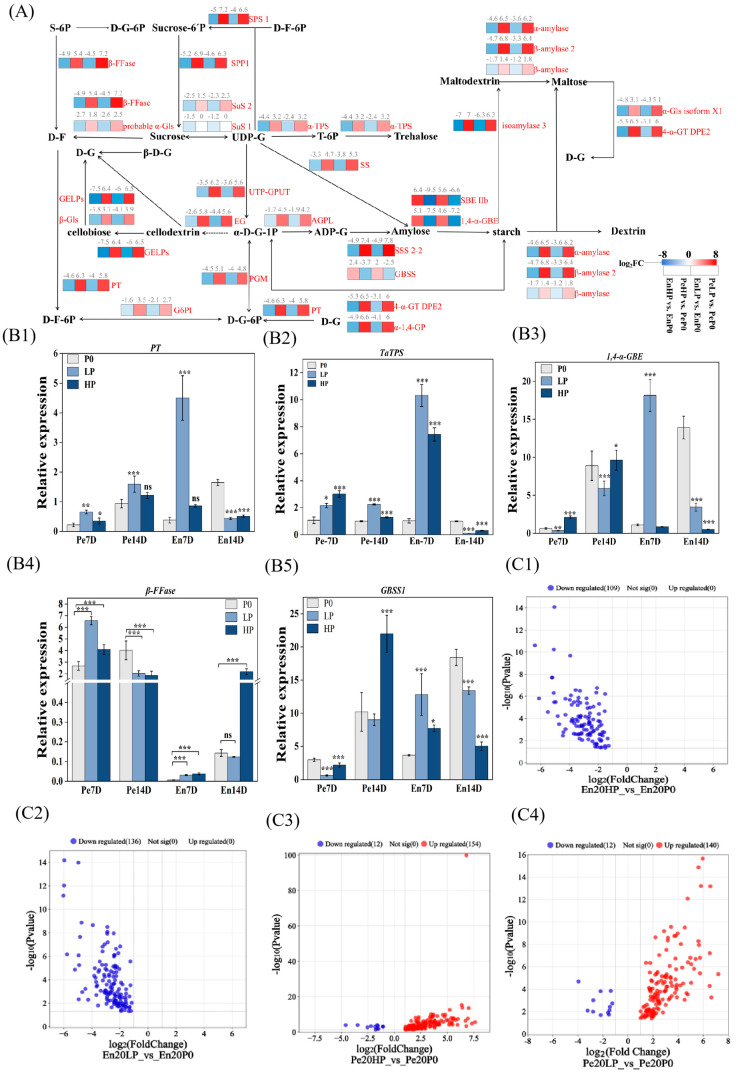
Phosphorus-regulated carbohydrate reallocation in wheat pericarp and endosperm. (**A**) Topological network analysis of starch-sucrose metabolism annotated with differentially expressed proteins (DEPs). Rectangular nodes depict protein expression dynamics under HP and LP treatments relative to P0, with log2(Fold Change) values encoded through a bichromatic gradient: red-to-blue spectrum represents upregulated-to-downregulated expression levels (see colorimetric scale). Numerical annotations above each node quantitatively specify log2(FC) magnitudes, while arrows represent the direction of carbon flow or metabolic conversions within the network. The topological architecture highlights rate-limiting enzymes and regulatory hubs governing starch biosynthesis (amylopectin/amylose branching) and sucrose mobilization pathways. This systems-level visualization identifies phosphorus-responsive modulators of carbon partitioning, particularly emphasizing DEPs associated with UDP-glucose pyrophosphorylase (UGPase) and sucrose synthase (SuSy) nodal activities. (**B1**–**B5**) Relative expression levels of genes encoding key enzymes in starch-sucrose metabolism. Data represent mean values ± SE (*n* = 3 biological replicates). Statistical significance was determined by *t*-tests (* *p* < 0.05, ** *p* < 0.01, *** *p* < 0.001; ns = not significant). (**C1**–**C4**) Proteomic profiles of DEPs associated with amino acid metabolism across phosphorus regimes. Comparative groups: (**C1**) En20HP vs. En20P0; (**C2**) En20LP vs. En20P0; (**C3**) Pe20HP vs. Pe20P0; (C4) Pe20LP vs. Pe20P0.

Wheat pericarp and endosperm exhibit compartment-specific metabolic reprogramming in response to differential phosphorus availability, orchestrating complex adjustments across central carbon metabolism pathways including glycolysis, pyruvate metabolism, TCA cycle, amino acid homeostasis, and starch-sucrose interconversion. Phosphorus supplementation triggered significant downregulation of endosperm metabolic enzymes, with three prominent exceptions: granule-bound starch synthase (GBSS), starch branching enzyme IIb (SBE IIb), and 1,4-α-glucan branching enzyme (1,4-α-GBE). This regulatory cascade extended to critical nodes in UDP-glucose metabolism, where sucrose synthase isoforms (SuS1/2) exhibited reduced expression, accompanied by coordinated suppression of glucose-6-phosphatase (G6PI) and β-amylase. Notably, pericarp SuS1 maintained constitutive expression across phosphorus regimes, standing in marked contrast to its endosperm counterpart, which showed phosphorus-dependent suppression (Figure 6A). These phosphorus-responsive proteomic shifts imply selective activation of starch biosynthetic apparatus, potentially favoring specific granule assembly pathways. Complementary qPCR profiling of associated metabolic genes (Figure 6(B1–B5)) uncovered intricate post-transcriptional regulatory dynamics. The observed transcriptional-metabolic dissociation underscores a hierarchical regulatory architecture coordinating phosphorus-responsive starch metabolism.

Amino acid metabolic pathways exhibited compartment-specific phosphorus responsiveness, revealing fundamental differences between grain tissues. Endosperm analysis demonstrated universal down-regulation of DEPs under both HP and LP relative to P0 (Figure 6(C1,C2)). In stark contrast, pericarp proteomes displayed pronounced phosphorus sensitivity, with HP vs. P0 comparisons identifying 166 DEPs (154 up-regulated) and LP vs. P0 analyses revealing 152 DEPs (140 upregulated) (Figure 6(C3,C4). This diametric regulation pattern-endosperm-wide suppression versus pericarp-specific activation-suggests tissue-differentiated metabolic strategies: phosphorus-mediated conservation of nitrogen resources in endosperm contrasted with proactive stress adaptation mechanisms in pericarp tissues.

### 2.7. Metabolic Adaptations in Pericarp and Endosperm Under Phosphorus Application: Insights from Starch Granule Proteomics

**Figure 7:**
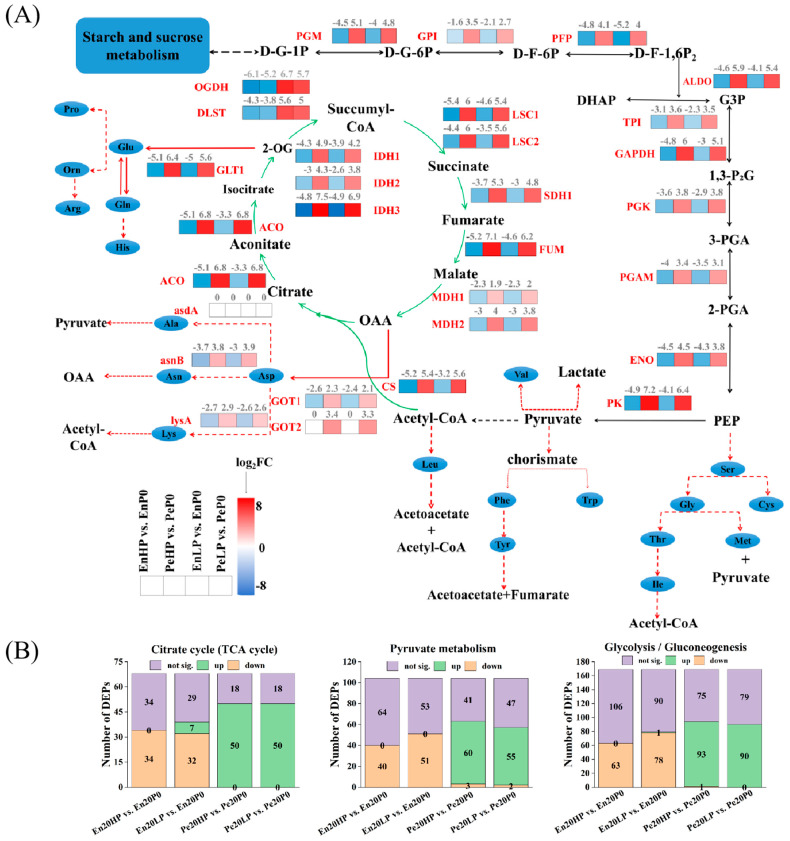
Schematic illustration of phosphorus-mediated the protein level changes in wheat pericarp and endosperm tissues. (**A**) The diagram delineates key metabolic pathways including glycolysis (black arrows), tricarboxylic acid (TCA) cycle (green arrows), pyruvate metabolism, and amino acid metabolism pathways (red arrows and the dashed line indicates that it needs to be achieved through multiple reactions). Differential protein expression patterns under HP and LP treatments are presented relative to P0. The direction of the arrow indicates the direction of metabolism, representing the direction of carbon flow or metabolic conversions within the network. The TCA cycle intermediates are particularly emphasized through green connectors, the glycolysis intermediates are particularly emphasized through black connectors, while critical nodes of amino acid anabolism are highlighted in red to demonstrate phosphorus-dependent metabolic channeling. This integrated representation reveals the systemic metabolic shift from energy production to biosynthetic processes under varying phosphorus availability. (**B**) Number of differentially expressed proteins in the TCA cycle, pyruvate metabolism, and Glycolysis/Gluconeogenesis pathway.

During the metabolism of starch and sucrose, Glucose-1-phosphate (D-G-1P) is produced and subsequently converted into pyruvate through glycolysis, which provides both energy and intermediate products. As the final product of glycolysis, pyruvate generates acetyl-CoA and enters the TCA cycle. Then, carbon skeletons of amino acids were integrated into the TCA pathway via various intermediates (Figure 7A). Complementary analysis of starch granule proteomics revealed that the DEPs in the pericarp were generally up-regulated, while those in the endosperm were generally down-regulated, after phosphorus application (Figure 7B). In summary, the observed differences in protein expression between the pericarp and endosperm may stem from variations in metabolic demands, functional roles, and environmental adaptations of these two tissues. The pericarp exhibited higher metabolic activity in response to increased energy needs and environmental stresses, whereas the endosperm tended to maintain a relatively low metabolic state, prioritizing material storage and supply.

## 3. Discussion

Our observations reveal that phosphorus application markedly induced the expansion of large amyloplast membranes and significantly increased the number of B-type starch granules (Figure 1). This phenomenon can be explained by the expanded membrane surface area, which provides additional sites for enzyme attachment and facilitates the generation of new small starch granules (SSGs) [22]. Furthermore, phosphorus application significantly enhanced the activities of SuSase and α-amylase, along with the content of soluble sugars (Figure 2), indicating a more active starch-sucrose metabolism. These findings collectively demonstrate that phosphorus facilitates starch granule accumulation by promoting amyloplast expansion and activating key enzymes in sucrose-starch conversion.

This physiological mechanism explains why extensive phosphorus fertilization has become a common practice to alleviate soil phosphorus stress and achieve high yields, given that wheat can only absorb a minimal portion of soil soluble phosphorus [23]. However, the environmental consequences of excessive phosphorus use—including rapid loss of soil organic matter [24] and water eutrophication—pose serious threats to ecosystem and human health, underscoring the need for balanced phosphorus management.

Our investigation into the starch granule-associated proteins (SGAPs) under differential phosphorus regimes reveals that wheat adapts to phosphorus levels through a systems-level reprogramming of multiple metabolic pathways. A pivotal discovery of our study is the diametrically opposed regulatory patterns between the pericarp and endosperm. This tissue-specific reprogramming suggests a “metabolic division of labor” within the grain: the pericarp, as a dynamic interface with the environment, employs metabolic plasticity for rapid response, whereas the endosperm prioritizes stable storage reserve accumulation. This paradigm is evidenced by the predominant upregulation of pericarp SGAPs contrasted with the marked downregulation in the endosperm (Figure 5 and Figure 6). Functional annotation and pathway enrichment analysis (Figure 5) confirmed the central role of glycolysis, pyruvate metabolism, TCA cycle, amino acid metabolism, and starch-sucrose metabolism in this process, aligning with previous reports where metabolic flexibility constitutes the primary adaptive mechanism to phosphorus stress [20,25].

At the enzymatic level, this divergence was exemplified by phosphorus supplementation enhancing SuSase activity and soluble sugar accumulation in the pericarp, while suppressing its expression yet paradoxically elevating its activity in the endosperm (Figure 2). This discrepancy between gene expression and enzyme activity may arise from post-translational modifications or the regulation of specific enzyme isoforms, a phenomenon observed in other complex plant systems [26]. The interplay between phosphorus and nitrogen metabolism further underscores the complexity of nutrient coordination. Amino acids, serving as fundamental transport vehicles for organic nitrogen [27], exhibited a distinct metabolic shift under phosphorus limitation. The higher content of most hydrolyzed amino acids in the P0 group, contrasted with the opposing trend for free amino acids (Figure 3), suggests that wheat employs protein catabolism as an adaptive strategy to mobilize nitrogen and carbon reserves. This aligns with the established paradigm of stress-induced metabolic reprogramming that optimizes the growth-defense balance [28,29,30].

The metabolic crosstalk extends to conserved biochemical nodes, with D-G-1P emerging as a critical nexus bridging starch-sucrose cycling and glycolytic pathways [31]. The significant down-regulation of glycolytic enzymes like glucose-6-phosphate isomerase (G6PI) and triosephosphate isomerase (TPI) in the endosperm, concurrent with their up-regulation in the pericarp under HP and LP treatments (Figure 6 and Figure 7), suggests a tissue-specific channeling of carbon flux. This inhibition of endosperm glycolysis at the D-G-1P level may indirectly promote amylose synthesis. Furthermore, the up-regulation of pathways associated with 3-phosphoglycerate synthesis in the pericarp likely accounts for the increased activity of AGPase—a key activator for this rate-limiting enzyme [32]—and facilitates A-type granule production [33]. The opposing differential expression of pyruvate kinase (PK) between tissues (up in pericarp, down in endosperm, Figure 7) further reinforces the “metabolic division of labor,” indicating a redirection of carbon away from the final step of glycolysis in the storage tissue (endosperm) towards anabolic processes.

In summary, our multi-faceted analysis demonstrates that wheat finely coordinates carbon allocation and starch biosynthesis under varying phosphorus supply through a tissue-specific “metabolic division of labor.” Phosphorus supply reprograms SGAP expression, accelerating starch turnover in the metabolically active pericarp while promoting storage compound synthesis in the endosperm. This shift ultimately enhances endosperm amylose synthesis and alters A/B-type starch granule differentiation. These insights into the distinct metabolic priorities of the pericarp and endosarm provide a theoretical foundation for precision fertilization strategies aimed at synergistically enhancing both wheat quality and yield.

## 4. Materials and Methods

### 4.1. Experimental Site and Experimental Design

The experiment was carried out from October 2021 to June 2022 at Shihezi University, Xinjiang, China (86.05 ′E, 44.31 ′N). The soil type was gray desert soil. The soil organic matter content was 1.54%, the alkaline hydrolyzable nitrogen content was 63 mg/kg, the available phosphorus content was 15 mg/kg, and the available potassium content was 208 mg/kg. Elite winter wheat *Triticum aestivum* L. cv. XinDong 20 (One of the main cultivars in Xinjiang, China) was used in this experiment. This experiment adopted a randomized complete block design. A detailed field layout and planting pattern are provided in Figure S3 (see Supplementary Materials). Three superphosphate (P0, 0 kg·ha^−1^, phosphorus deficiency; LP, 105 kg·ha^−1^, normal phosphorus and HP, 210 kg·ha^−1^, phosphorus excess) were designed. Phosphorus was supplied as granular triple superphosphate (brand: ThreeCircles; total P_2_O_5_ ≥ 46.0%; available P_2_O_5_ ≥ 44.0%; conforming to GB21634-2008). The fertilizer was applied in two splits: 50% as basal fertilizer in bands at sowing, and the remaining 50% as topdressing in furrows at the reviving stage (160 days after sowing). Each treatment was replicated three times, with individual plot dimensions of 2.4 m × 3 m and a spacing of 50 cm between plots. Drip irrigation was used throughout the experiment. Before sowing, 75 kg·ha^−1^ of urea was applied. Additional urea was supplied via irrigation water at the jointing (45 kg·ha^−1^), heading (75 kg·ha^−1^), and flowering (120 kg·ha^−1^) stages. Wheat plants were irrigated three times before winter and six times from the reviving stage to maturity at 10–12 day intervals. Ears that flowered synchronously were labeled during the flowering stage.

### 4.2. Observation of Starch Morphology

Embryo-excised wheat grains (7 and 21 DPA, −80 °C stored) were transversely sectioned (about 2 mm), rinsed with PBS (0.1 M, pH 7.4), fixed in 2.5% glutaraldehyde (4 °C, 24 h), then ethanol gradient-dehydrated (30 min per step), tert-butanol-immersed (3 × 30 min), and freeze-dried (TIANFENG, TF-FD-1L, Shanghai, China). Pericarp (7 DPA) and endosperm (21 DPA) samples were imaged using SEM (HITACHI, SU8010, Tokyo, Japan) [34].

Glutaraldehyde-dimethyl arsenate-fixed grains (pH 7.0) were sliced (about 1 mm), processed by dehydration-infiltration-embedding [35], and ultrathin-sectioned (Leica, Ultracut R, Wetzlar, Germany). Uranyl acetate (30 min)- and lead citrate (15 min)-stained sections were analyzed using TEM (HITACHI, HT7700, Tokyo, Japan).

### 4.3. Measurements of Starch Granule Size Distribution and Amylopectin Chain Length Distribution

The starch extraction protocol was adapted from Li et al. [34]. Briefly, caryopses were degermed, mechanically disrupted, and soaked in aqueous solution. The crude starch was subsequently purified by differential centrifugation, during which low-density cellular components and surface proteins were removed using an 80% (*w*/*v*) CsCl gradient centrifugation step. All extraction procedures were performed with three biological replicates. The granule size distribution was determined by laser diffraction (Beckman Coulter LS 13320, Brea, CA, USA).

The amylopectin chain length distribution was assessed via high-performance anion-exchange chromatography with pulsed amperometry (HPAEC-PAD), following the methodology of Li et al. [35]. Each sample was analyzed in three biological replicates. Chains were categorized by the degree of polymerization (DP) into short (DP 6–18, A-chains), medium (DP 19–34, B1-chains), and long (DP > 35, B2-chains).

### 4.4. Determination of Starch and Soluble Sugar Content

Starch quantification was performed using the iodometric method [34]. Standard curves for amylose and soluble starch were constructed for quantification. The samples were treated sequentially with anhydrous ethanol and 1 M NaOH. After adding 1 M acetic acid and iodine reagent (0.2% I_2_, 2% KI), the reactions were incubated for 10 min. Absorbance at 620 nm was measured using a water blank. Amylose and total starch content were calculated from standard curves, with amylopectin content determined by subtracting amylose from total starch. Each sample was analyzed in three biological replicates.

Soluble sugar quantification was performed using anthrone-sulfuric acid colorimetry [36] with three biological replicates. Absorbance measurements at 620 nm were conducted using a UV-Vis spectrophotometer GENESYS™ 140 (Thermo Fisher Scientific, Waltham, MA, USA), with glucose standard solutions used to establish the calibration curve. Sugar concentrations were determined by linear regression analysis of absorbance values.

### 4.5. Sucrose Synthase (SuSy) Assay

The SuSy (EC 2.4.1.13) activity was quantified according to the protocol described by Zhang et al. [37] with three biological replicates. Fresh tissue samples (0.1–0.3 g) from the pericarp and endosperm were homogenized in ice-cold 50 mM HEPES-NaOH extraction buffer (pH 7.5) to obtain the crude enzyme extracts. The enzymatic a reaction was initiated by combining equal volumes of tissue homogenate and reaction solution containing 100 mM HEPES-NaOH (pH 7.5), 50 mM MgCl_2_, 100 mM UDP-glucose (UDPG), and 100 mM fructose. After incubation at 30 °C for 30 min, 200 μL of 2 M NaOH, 1.5 mL concentrated HCl, and 0.5 mL of a 0.1% resorcinol solution were added, mixed, and heated at 80 °C for 10 min. The sample was cooled to room temperature, and absorbance was measured at 480 nm using a microplate reader Infinite 200 PRO (TECAN, Männedorf, Switzerland). 

### 4.6. Determination of ADP-Glucose Pyrophosphorylase (AGPase) and Soluble Starch Synthase (SSS) Activity

The enzymatic activities of AGPase (EC 2.7.7.27) and SSS (EC 2.4.1.21) in pericarp and endosperm tissues were assayed following the methodology of Mahla et al. [38] with three biological replicates. Crude enzyme extracts were prepared by homogenizing fresh tissues in 1 mL of ice-cold extraction buffer containing 100 mM HEPES-NaOH (pH 7.5), 10 mM MgCl_2_, 2 mM EDTA, 50 mM β-mercaptoethanol, 12.5% (*v*/*v*) glycerol, and 5% (*w*/*v*) polyvinylpyrrolidone-40 (PVP-40). For activity measurements, the reaction mixture containing the enzyme extract was incubated under specified conditions. Absorbance readings were subsequently recorded at dual wavelengths (340 nm and 520 nm) using a UV-Vis spectrophotometer GENESYS™ 140 (Thermo Fisher Scientific, Waltham, MA, USA), with 340 nm monitoring NADPH oxidation for AGPase activity and 520 nm quantifying starch-iodine complex formation for SSS activity.

### 4.7. Determination of Starch-Degrading Enzyme Activity

According to the methodology established by Zhang [39], the enzymatic activities of α-amylase (EC 3.2.1.1) and β-amylase (EC 3.2.1.2) were determined with three biological replicates. Briefly, 2 mL of distilled water was added to the crude enzyme extract. A standard curve was constructed using the DNS (3,5-dinitrosalicylic acid) method, which involved DNS reagent (containing 2 M NaOH and 30 g potassium sodium tartrate), 1% (*w*/*v*) starch solution, and a series of maltose standard solutions (0–0.9 mg/mL). Absorbance was measured at 540 nm using a spectrophotometer, and the enzyme activities were calculated based on the standard curve.

### 4.8. Determination of Amino Acid Content

Wheat grains of 7 and 14 DPA were ground, mixed with 0.02 M HCl (final volume: 10 mL), and centrifuged. A 2.5 mL aliquot was combined with 1.5 mL 0.02 M HCl, purified via C18 column (pre-activated with 5 mL methanol/water), adjusted to 5 mL with 0.02 M HCl, filtered (0.45 µm), and analyzed triplicate using an amino acid analyzer (HITACHI, LA8080, Tokyo, Japan) for 17 free amino acids [36].

For 35 DPA wheat grains (200 mg dry weight), acid hydrolysis was performed according to Bai et al. [40] using 6 M HCl containing 0.1% phenol at 110 ± 1 °C for 22 h. The hydrolysate was vacuum-filtered, evaporated to dryness under nitrogen gas, reconstituted in pH 2.2 sodium citrate buffer, passed through a 0.22 μm syringe filter, and analyzed in triplicate using the LA8080 system (HITACHI, Tokyo, Japan). Each sample was analyzed in three biological replicates.

### 4.9. Determination of Phosphorus Content and Acid Phosphatase (APase) Activity

Endosperm and pericarp samples were dried, ground, and digested (0.2 g) with HNO_3_-HClO_4_-H_2_SO_4_ (26:3.2:0.8 mL) at 230–300 °C, respectively [41]. A 5 mL aliquot was mixed with chromogenic reagent (10% (NH_4_)_6_Mo_7_O_24_·4H_2_O, 0.5% NH_4_VO_3_ in 35% HNO_3_ matrix), and absorbance measured at 460 nm. Each sample was analyzed in three biological replicates.

Powdered tissues (0.1 g) were homogenized in 50 mM sodium acetate buffer (pH 6.0), centrifuged (13,000× *g*, 10 min), and incubated with 5 mM p-nitrophenyl phosphate (PNPP) at 28 °C [42] with three biological replicates. Reactions were terminated with 1 M NaOH after 30 min, centrifuged, and the absorbance was read at 405 nm against a buffer blank.

### 4.10. RNA Extraction and Determination of Gene Expression

Total RNA was isolated from pericarp and endosperm tissues using the EASYspin Plus Plant RNA Kit (Aidlab, Cat # RN3802, Beijing, China). First-strand cDNA synthesis was using performed the EasyScript^®^ One-Step gDNA Removal and cDNA Synthesis SuperMix (TransGen, Cat# AE311, Beijing, China), according to the manufacturer’s protocol. Quantitative real-time PCR (qRT-PCR) was performed using a Roche LightCycler 480 II system (Basel, Switzerland). Gene expression fold-changes were calculated using the 2^−ΔΔCt^ method, with *β-actin* (Gene ID: DN551593) serving as the internal reference gene [43]. Each experimental group consisted of three biological replicates. Primer sequences (Table S1) were designed using NCBI Primer-BLAST and synthesized by Sangon Biotech (Shanghai, China).

### 4.11. Extraction of Starch Granule-Associated Proteins and Quantitative Proteomic Profiling with Data-Independent Acquisition (DIA)

Wheat embryos (7 and 14 DPA) were dissected, and pericarp/endosperm tissues were separated. Starch granules were isolated as described by Yu and Wang [44], with modifications. Tissues were homogenized in ice-cold buffer (50 mM Tris-HCl pH 8.0, 1 mM EDTA, and 2 mM PMSF), nylon-strained (250 mesh), and then centrifuged (500× *g*, 30 min, 4 °C) over 100% Percoll. Pellets were washed seven times with wash buffer (Tris-HCl/EDTA/PMSF with 10% glycerol and 0.2% Triton X-100), three ultrapure water rinses, two acetone washes, and air-drying. For protein extraction, granules (25 mg/mL) were treated with 0.01 M NaOH (25 °C, 15 min), centrifuged (4000× *g*, 5 min), and neutralized to a stable pH through iterative water washing.

SGAPs were extracted using the method of Bancel et al. [45] with modifications: 0.3 g starch was boiled (100 °C, 10 min) in lysis buffer (62.5 mM Tris-HCl pH 8.7, 2% SDS, 10 mM DTT), ice-cooled (10 min), and centrifuged (10,000× *g*, 10 min). The supernatants were acetone-precipitated (20% TCA, −20 °C overnight), centrifuged (12,000× *g*, 20 min), washed with 80% acetone, air-dried, and dissolved in Tris-sucrose-SDS buffer. Phenol-phase separation and methanol precipitation (0.1 M NH_4_Ac in methanol, −20 °C overnight) yielded purified proteins. Protein concentration was determined using the Bradford assay (595 nm), and the quality was verified via SDS-PAGE.

DIA analysis (Shenzhen BGI) used MaxQuant-processed SWATH-MS data (FDR ≤ 1%). Protein identification was performed using a target-decoy library, with DEPs defined as a ≥2-fold change (*p* < 0.05) at FDR < 1%.

### 4.12. Data Analysis

Statistical analyses were performed using SPSS 26.0 (IBM Corporation, Armonk, NY, USA). Independent sample *t*-tests were conducted to evaluate significant differences between the P0 and LP/HP groups. For comparisons among multiple groups at the same developmental stage, one-way analysis of variance (ANOVA) was employed. Graphical representations were generated using Origin 2025b (OriginLab Corporation, Northampton, MA, USA). Volcano plots, Kyoto Encyclopedia of Genes and Genomes (KEGG) enrichment dot bubbles, and Gene Ontology (GO) enrichment circles were created using the bioinformatics online platform, a web-based data analysis and visualization tool. The URL should be cited with the access date as follows: https://www.bioinformatics.com.cn (accessed on 23 May 2024).

## 5. Conclusions

In conclusion, this study provides novel insights into the mechanistic role of phosphorus in modulating source–sink carbon partitioning in wheat. Our results clearly demonstrate the superiority of phosphorus fertilization over the non-phosphorus control in driving key physiological processes. Phosphorus application was markedly superior in promoting the expansion of amyloplast membranes and the formation of B-type starch granules, while also significantly enhancing the activities of SuSy and α-amylase and elevating soluble sugar content compared to P-deficient conditions. More importantly, we reveal that phosphorus orchestrates a compensatory metabolic interplay between the pericarp and endosperm: it enhances starch degradation in the pericarp (source tissue) to fuel starch synthesis and accumulation in the endosperm (sink tissue). This metabolic reprogramming was substantiated by proteomic evidence showing that phosphorus application induced more up-regulated DEPs in pericarp starch granule proteins, whereas endosperm DEPs were predominantly down-regulated.

These findings validate the agronomic necessity of phosphorus fertilization for wheat cultivation, moving beyond mere yield enhancement to include qualitative improvements in grain development. The demonstrated superiority of phosphorus in regulating these essential metabolic pathways underscores its irreplaceable role in sustainable wheat production. Future research should focus on elucidating the molecular regulators governing this phosphorus-mediated source–sink communication, particularly the key enzymes and signaling pathways involved in inter-tissue carbon allocation. Such investigation will provide crucial targets for developing improved wheat varieties with enhanced phosphorus utilization efficiency.

## Data Availability

The data reported in this paper have been deposited in the OMIX, China National Center for Bioinformation/Beijing Institute of Genomics, Chinese Academy of Sciences (https://ngdc.cncb.ac.cn/omix (accessed on 5 August 2025): accession no.OMIX011308).

## Supplementary Materials

The following supporting information can be downloaded at: https://www.mdpi.com/article/10.3390/plants14203152/s1, Figure S1: Phosphorus-dependent modulation of starch granule development and amylopectin fine structure in wheat grains; Figure S2: The number of differentially expressed proteins (DEPs) under different phosphorus supply levels; Figure S3: Experimental planting diagram; Table S1: Primer sequences for real-time PCR.

## Abbreviations

SPAGs	starch granule-associated proteins
KEGG	Kyoto Encyclopedia of Genes and Genomes
GO	Gene Ontology
DPA	days post-anthesis
1,3-P_2_G	1,3-bisphosphoglyceric acid
1,4-GBE	1,4-alpha-glucan-branching enzyme
2-OG	α-ketoglutaric acid
2-PGA	2-phosphoglyceric acid
3-PGA	3-phosphoglyceric acid
ACO	Aconitase
ADP-G	Adenosine Diphosphate Glucose
AGPase	ADP-Glucose Pyrophosphorylase
AGPL	Glucose-1-phosphate adenylyltransferase
ALDO	Aldolase
asdA	Aspartate Semialdehyde Dehydrogenase A
asdB	Aspartate Semialdehyde Dehydrogenase B
CS	Citrate synthase
DEPs	differentially expressed proteins
D-F-1,6P2	D-fructose-1,6-diphosphate
D-F-6P	D-Fructose-6 phosphate
D-G	D-Glucose
D-G-1P	D-Glucose-1 phosphate
D-G-6P	D-Glucose-6 phosphate
DHAP	dihydroxyacetone phosphate
DLST	Dihydrolipoamide Succinyltransferase
EG	Endoglucanase
ENO	Enolase
FUM	Fumarase
G3P	glyceraldehyde-3-phosphate
G6PI	Glucose-6-phosphate isomerase 6
GAPDH	Glyceraldehyde-3-phosphate dehydrogenase
GBSS	Granule-bound Starch synthase
GELPs	GDSL esterase/lipase
GLT	Glutamate Synthase
GOT	Glutamate Oxaloacetate Transaminase
GPI	Glucose-6-phosphate isomerase
IDH	Isocitrate Dehydrogenase
LSC	Succinyl—CoA Ligase
lysA	Diaminopimelate Decarboxylase
MDH	Malate Dehydrogenase
OAA	Oxaloacetic acid
OGOH	α-Ketoglutarate Dehydrogenase
PEP	Phosphoenol-pyruvate
PFP	Fructose 6-phosphate 1-phosphotransferase 6
PGAM	Phosphoglycerate mutase
PGK	Phosphoglycerate kinase
PGM	Phosphoglucomutase
PK	pyruvate kinase
PT	Phosphotransferase
S-6P	Sucrose-6 phosphate
SBE	Starch branching enzyme
SBE IIb	starch branching enzyme IIb
SDH	Succinate Dehydrogenase
SEM	Scanning electron micrographs
SGAPs	starch granule-associated proteins
SPS	Sucrose-phosphate synthase
SS	Starch synthase
SSGs	small starch granules
SSS	Soluble starch synthase
SuSy	Sucrose synthase
T-6P	Trehalose-6 phosphate
TEM	Transmission electron micrographs
TPI	Triosephosphate isomerase
UDP-G	Uridine Diphosphate Glucose
UTP-GPUT	UTP-glucose-1-phosphate uridylyltransferase
α-1,4-GP	Alpha-1,4-glucan phosphorylase
α-TPS	Alpha-trehalose-phosphate synthase
β-FFase	Beta-fructofuranosidase
β-Gls	Beta-glucosidase
