# Supplementary material for: Phosphorus Alters the Metabolism of Sugars and Amino Acids in Elite Wheat Grains
Source: Plants (Basel). 2025 Oct 13;14(20):3152. doi: 10.3390/plants14203152 (PMC12566986; doi:10.3390/plants14203152)
Supplement: Supplementary file 1 [file plants-14-03152-s001.zip › plants-3889694-supplementary.pdf]

Table S1. Primer sequences for real-time PCR

Primers	Sequences（5'-3')
PHT2-F	TGGCCCTAGAGGAGCTCAAT
PHT2-R	AAAACCACCCCAAGCAAGGA
GBSS1-F	GACACTATCGTGGAAGGCAAG
GBSS1-R	TTGACCATCTCATGGTACGC
α-TPS-F	TGAGTGCGTCGTTGTTACTGC
α-TPS-R	TTGTATCAATGTTCCAAGGGT
1,4-α-GBE-F	CGTTCGCAGGCTTTGTTGATAC
1,4-α-GBE-R	TGATGTCCACCTTGCCGTTGT
PT-F	ATGTCTGCCATGCACCACGAT
PT-R	TGTCCCGGCCTAGCTTCTTCA
β-FFase -F	CGTTCGCAGGCTTTGTTGATAC
β-FFase -R	TGATGTCCACCTTGCCGTTGT
actin-F	GGAAAAGTGCAGAGAGACACG
actin-R	TACAGTGTCTGGATCGGTGGT

Figure S1

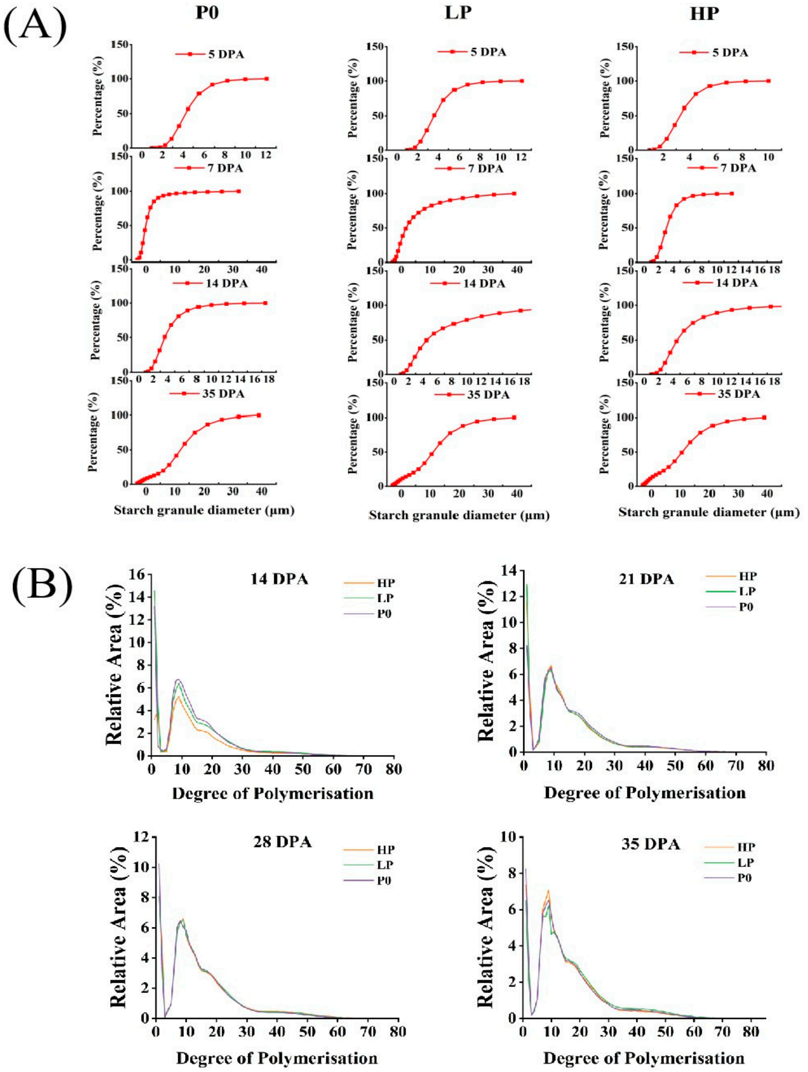

Supplementary Figure S1. Phosphorus-dependent modulation of starch granule development and amylopectin fine structure in wheat grains. (A) Temporal dynamics of starch granule size distribution during key developmental stages (5, 7, 14, 35 days post-anthesis, DPA) under differential phosphorus regimes: P0 (0 kg·ha^{-1}), LP (105 kg·ha^{-1}), HP (210 kg·ha^{-1}). Data presented as mean ± SD (n = 3 biological replicates); (B) Developmental-stage-specific modifications in amylopectin chain-length profiles. Chains were categorized as: DP 6-18 (A-chains); DP 19-34 (B1-chains); DP >35 (B2-chains).

Figures S2

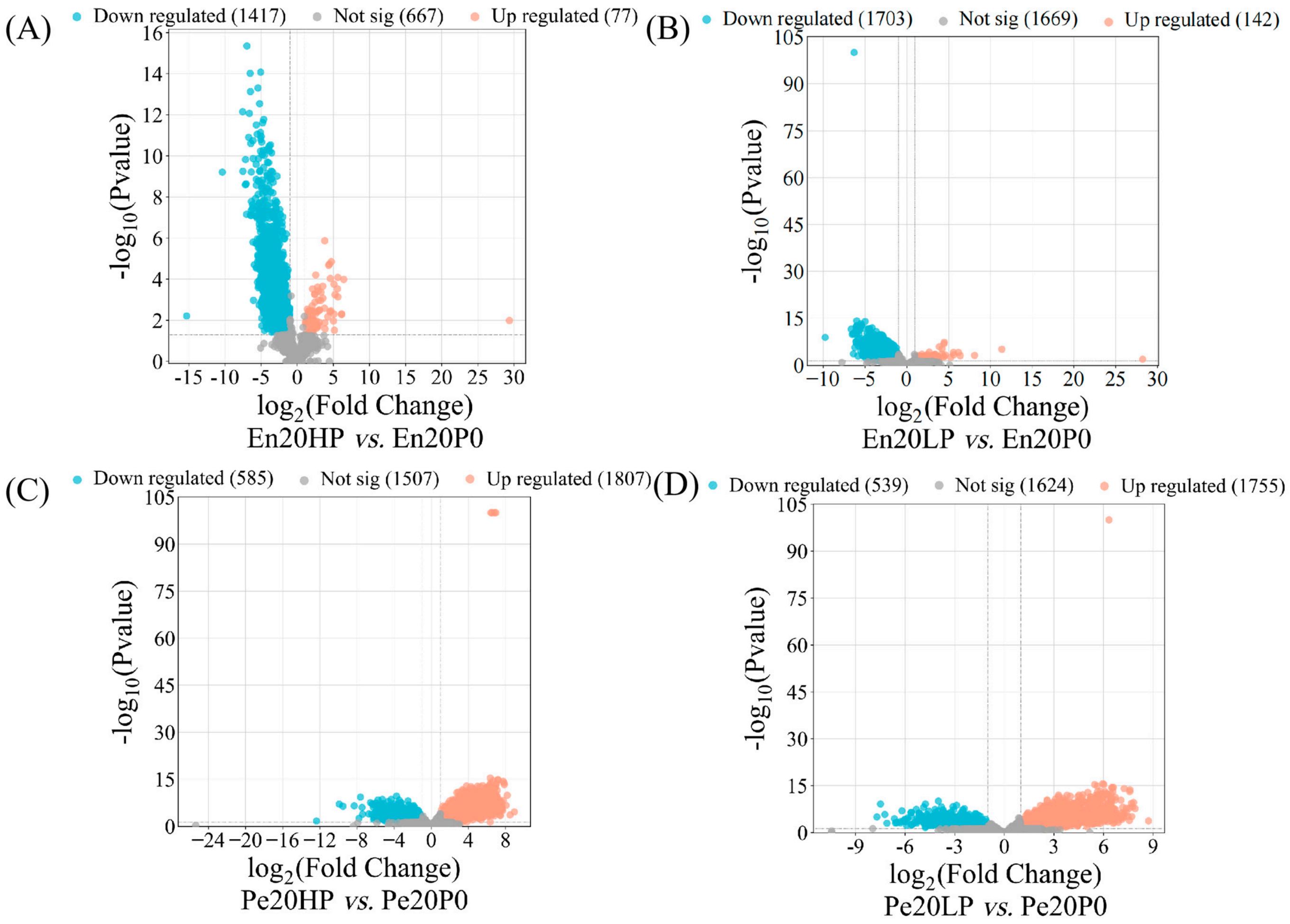

Supplmentary Figure S2. The number of differentially expressed proteins (DEPs) under different phosphorus supply levels. (A-D) The number of down-regulated and up-regulated DEPs in pericarp and endosperm starch granules under different phosphorus supply levels. Comparative groups: (A) EnHP vs. EnP0; (B) EnLP vs. EnP0; (C) PeHP vs. PeP0; (D) PeLP vs. PeP0.

Figure S3

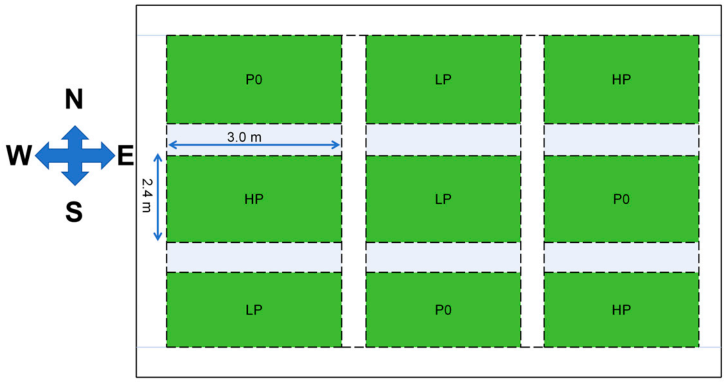

Supplementary Figure S3. Experimental planting diagram. Phosphorus regimes: P0 (0 kg·ha^{-1}), LP (105 kg·ha^{-1}), HP (210 kg·ha^{-1}).
